# TFEB-driven autophagy potentiates TGF-β induced migration in pancreatic cancer cells

**DOI:** 10.1186/s13046-019-1343-4

**Published:** 2019-08-06

**Authors:** Ruizhi He, Min Wang, Chunle Zhao, Ming Shen, Yahong Yu, Li He, Yan Zhao, Hua Chen, Xiuhui Shi, Min Zhou, Shutao Pan, Yuhui Liu, Xingjun Guo, Xu Li, Renyi Qin

**Affiliations:** 1 Department of Biliary-Pancreatic Surgery, Affiliated Tongji Hospital, Tongji Medical College, Huazhong University of Science and Technology, No.1095 Jie Fang Avenue, Wuhan, 430030 China; 2 Department of Trauma Surgery, Affiliated Tongji Hospital, Tongji Medical College, Huazhong University of Science and Technology, Wuhan, 430030 China

**Keywords:** Autophagy, TFEB, Endocytosis, Focal adhesion, Pancreatic cancer

## Abstract

**Background:**

Pancreatic ductal adenocarcinoma is one of the most aggressive cancers, with a 5-year survival rate of less than 8%. The complicated tumor microenvironment, particularly TGF-β, provides possible convenience for the progression of PC cells. TGF-β regulates critical cellular processes, including autophagy. However, the mechanism and effects of TGF-β-mediated autophagy are still poorly understood.

**Methods:**

Bioinformatics analysis, western blot, transmission electron microscopy and confocal microscopy were used to identify that TFEB is the key factors in TGF-β-induced autophagy. The biological effects of TFEB-driven autophagy were investigated in vitro using transwell and wound healing assays and in vivo using liver metastasis and *LSL-KrasG12D/Pdx1-Cre* mice models. Luciferase assays and motif analysis were used to assess regulation of RAB5A gene promoter activity by TGF-β-induced TFEB. TFEB levels were measured by real-time PCR, western blot and immunohistochemical staining in clinical pancreatic ductal adenocarcinoma tissues.

**Results:**

We demonstrated that TGF-β induces TFEB expression via the canonical smad pathway in Smad4-positive PC cells and facilitates TFEB-mediated autophagic activation. TFEB-driven autophagy caused by TGF-β regulates RAB5A-dependent endocytosis of Itgα5 and promotes progression of PC cells. We further showed that enhanced TFEB expression and its direct target RAB5A both predict poor prognosis in PC patients.

**Conclusions:**

Our findings reveal TFEB-driven autophagy is required for TGF-β induced migration and metastasis of PC cells by promoting endocytosis of Itgα5β1 and focal adhesion disassembly through the TGF-β-TFEB-RAB5A axis. Our results highlight the potential utility of suppressing TFEB-driven autophagy to block PC metastasis.

**Electronic supplementary material:**

The online version of this article (10.1186/s13046-019-1343-4) contains supplementary material, which is available to authorized users.

## Background

With a 5-year survival rate less than 8%, pancreatic ductal adenocarcinoma is the seventh leading cause of cancer death due to its poor prognosis [1]. Many studies have demonstrated that the tumor microenvironment, including interstitial cells and proliferated interstitial components, plays a significant role in pancreatic cancer (PC) cell survival and metastatic dissemination [2, 3]. Proliferation of fibrotic tissue is stimulated by multiple cytokines, such as paracrine transforming growth factor-β (TGF-β), which regulates diverse cellular biological processes [2, 4–6]. TGF-β acts as a tumor suppressor in early stage tumors but paradoxically functions as a potent tumor promoter in advanced cancers [7]. Previous studies have shown that TGF-β promotes the invasion and metastasis of PC through multiple mechanisms [4, 8–10]. However, the precise mechanism underlying TGF-β-induced invasion in cancer has not been fully elucidated and requires further exploration.

Autophagy is an evolutionarily conserved catabolic process that was originally recognized as a crucial pro-survival mechanism that supplies the cell with nutrients under unfavorable grown conditions [11]. During autophagy, the subcellular membranes sequester a portion of cytoplasm and organelles for lysosomal degradation [12]. Several studies have demonstrated an involvement of dysregulated autophagy in many human diseases, including cancers [13]. Although genetically engineered mouse models demonstrated autophagy prevents normal cells from transforming into primary tumor cells, cancer cells still rely on autophagy to maintain survival [14]. Recent studies have shown that autophagy plays a critical role in promoting metastasis in multiple types of pre-existing cancers [15, 16]. TGF-β has been reported to regulate autophagy in some cancer cells [17–19]. However, the mechanism and effects of TGF-β-mediated induction of autophagy in cancer are largely unclear.

The transcription factor EB (TFEB) is a central regulator of the autophagic process that functions by regulating the CLEAR (coordinated lysosomal expression and regulation) gene network [20–22]. Here we report that TFEB is induced during TGF-β treatment via the canonical TGF-β/smad pathway. We demonstrate that TGF-β treatment leads to TFEB-driven autophagy, which is required for the migration and metastasis of PC cells due to TFEB-mediated endocytosis of Itgα5β1 and focal adhesion disassembly via transcriptional activating the RAB5A gene controlled by the CLEAR network. Importantly, up-regulation of TFEB was associated with shorter survival in PC patients. These results broaden our understanding of autophagy and pancreatic cancer progression.

## Methods

### Cell lines and reagents

MIA PaCa-2, SW1990, CFPAC-1 and PANC-1 were purchased from American Type Culture Collection (Manassas, VA, USA); AsPC-1, BxPC-3 and Panc 03.27 were purchased from the Cell Repository, Chinese Academy of Sciences (Shanghai, China). The immortalized human pancreatic ductal epithelial (HPDE) cell line was obtained from Beijing North Carolina Chuanglian Biotechnology Research Institute (Beijing, China). MIA PaCa-2, CFPAC-1 and PANC-1 were cultured in DMEM (Gibco) and SW1990, AsPC-1, BxPC-3, Panc 03.27 and HPDE were cultured in 1640 medium (Gibco) supplemented with 10% fetal bovine serum (FBS) in a 5% CO_2_ atmosphere at 37 °C. The following antibodies and chemical reagents were purchased: anti-LC3B (3868), anti-SQSTM1/p62 (8025), anti-TFEB (37785), anti-phospho-FAK (Tyr397) (8556), anti-Smad2/3 (8685), anti-Smad4 (38454), anti-phospho-Smad2(Ser465/467) (3108), anti-phospho-p44/42 MAPK (Erk1/2) (Thr202/Tyr204) (4370), anti-Phospho-p38 MAPK (Thr180/Tyr182) (4511) and Integrin Antibody Sampler Kit (4749) were obtained from Cell Signaling Technology (Beverly, MA, USA); anti-GAPDH (60004–1-Ig), anti-TFEB (13372–1-AP), anti-Histone-H3 (17168–1-AP) and anti-RAB5A (66339–1-Ig) were obtained from Proteintech Group (Chicago, IL, USA); anti-CTSD (ab75852) was purchased from Abcam; anti-LAMP2 (sc-18,822) was obtained from Santa Cruz Biotechnology (Santa Cruz, CA, USA); anti-Smad2/3 (AF3797) and anti-Zyxin (NBP1–90349) were obtained from Novus Biologicals (Littleton, CO, USA); anti-Paxillin (612405) was obtained from BD Biosciences (San Jose, CA, USA); anti-LAMP1 (sc-18,821) was obtained from Santa Cruz Biotechnology (Dallas, TX, USA); Sulfo-NHS-SS-Biotin (21331) was obtained from ThermoFisher (Shanghai, China). MesNa (1392807), iodoacetamide (I114), alantolactone (SML0415), chloroquine (CQ; C6628) and nocodazole (M1404) were obtained from Sigma-Aldrich (St. Louis, MO, USA); recombinant human TGF-β (100-21C) was obtained from Peprotech (Rocky Hill, NJ, USA); recombinant mouse TGF-β (7666-MB) was obtained from R&D (Minneapolis, MN, USA). LY364947 (S2805) was obtained from Selleck (Houston, TX, USA).

### Western blot analysis

An equivalent amount of protein sample was separated by SDS-PAGE gel and then transferred onto PVDF membrane (Millipore). After incubation with primary antibodies overnight, followed by incubation with specific secondary antibodies coupled to horseradish peroxidase, the blots were then detected with a ChemiDoc XRS System (Bio-Rad Laboratories). For total cellular protein isolation, cells or tissue samples were harvested and lysed on ice in RIPA buffer with protease inhibitor cocktail for 30 min. After centrifugation at 12,000 g for 15 min, the supernatant was collected as total cellular protein extract. Nuclear protein and cytoplasmic protein were isolated using subcellular nuclear and cytosolic protein extraction kit (Boster Biological Technology, Wuhan, China) according to the manufacturer’s specifications. Protein concentration was measured by the bicinchoninic acid protein assay kit (Beyotime, China).

### Transmission electron microscopy

Cells were fixed in 2.5% glutaraldehyde in 0.1 M cacodylate buffer at 4 °C overnight after the indicated treatments, and then postfixed with 1% osmium tetroxide in 0.1 M cacodylate buffer for 1 h at 4 °C. After dehydration through a graded series of ethanol, the cells were embedded in spur resin. Serial sections were cut with an ultramicrotome and stained with 4% uranyl acetate and lead citrate. Images were captured by a Hitachi H-7000FA transmission electron microscope.

### Gene silencing with use of shRNA and transfection

The specific TFEB shRNA target was 5′ CCGGGAAAGGAGACGAAGGTTCAACCTCGAGGTTGAACC TTCGTCTCCTTTCTTTTTG 3′.The specific ATG5 shRNA target was 5′ CCGGAAGTGAGATATGGTTTGAAT ACTCGAGTATTCAAACCATATCTCACTTTTTTTG 3′. The lentiviral vector containing the GFP-LC3B reporter, TGF-β and tandem-labeled GFP-mRFP-LC3 reporter were constructed by GenePharma (Shanghai, China), and transfection was implemented according to the manufacturer’s specification respectively. The specific small interfering RNA targeting Smad4 and the corresponding control were chemically synthesized by Ribobio (Guangzhou, China). Transfection of plasmid was carried out using Lipofectamine 2000 (Invitrogen) according to the manufacturer’s instructions. Forty-eight hours after transfection, the cells were incubated with the indicated reagents for further experiments.

### Plasmid construction and luciferase reporter assays

To generate the CLEAR site report plasmid, fragments of 6 × CLEAR sites were cloned into the XhoI-HindIII site of pGL3 vector, and pRL-TK was a kind gift from Cancer Research Institute, Tongji Hospital. After co-transfected with 1μg CLEAR site pGL3 and 0.2μg pRL-TK in cells for 48 h, luciferase activity was detected with the Dual-Luciferase Reporter Assay System (Promega, Madison, WI, USA) according to the manufacturer’s instructions. The relative luciferase activity was determined by a GloMax 20/20 Luminometer (Promega). Luciferase activity was normalized to firefly luciferase activity.

### Immunofluorescence and confocal microscopy

For immunofluorescence analysis, cells were seeded on glass coverslips placed in 6-well plates. Following the indicated treatments, cells were fixed with 4% formaldehyde for 30 min and permeabilized with 0.1% Triton X-100 (Sigma-Aldrich) for 10 min. Fixed cells were incubated with primary antibody against primary at 4 °C overnight after being blocked with 5% BSA for 30 min at room temperature. The cells were then incubated with Cy3/FITC-conjugated secondary antibody for 1 h at 37 °C and counterstained with DAPI (Sigma-Aldrich) for 10 min. Cells were subsequently visualized under a confocal microscope LSM710 (Carl Zeiss, Germany, LSM710).

Cells transfected with GFP-LC3B or GFP-mRFP-LC3B were grown on glass coverslips. Following the designated treatments, cells were fixed with 4% formaldehyde for 30 min and photographed using a confocal microscope (Carl Zeiss, Germany, LSM710).

### Real-time PCR

Surgical samples of pancreatic cancers and matching none-tumor tissues were obtained from Tongji Hospital for RNA extraction. Total RNA was extracted from pancreatic cancer cells as indicated or tissue samples using a TRIzol kit (Invitrogen) according to the manufacturer’s instructions. For cDNA synthesis, equal amounts of RNA were transcribed, and random primers (TAKARA) were used for reverse transcription as described previously. Gene expression was measured on an ABI StepOnePlus using SYBRGreen (TAKARA). The housekeeping gene GAPDH was used as reference gene in all RT-qPCR analyses. The forward primer for TFEB was 5′- ACCTGTCCGAGACCTATGGG − 3′, the reverse primer for TFEB was 5′- CGTCCAGACGCATAATGTTGTC − 3′, the forward primer for LAMP-1 was 5′- TCTCAGTGAACTACGACACCA − 3′, the reverse primer for LAMP-1 was 5′- AGTGTATGTCCTCTTCCAAAAGC − 3′, the forward primer for CTSD was 5′- ATTCAGGGCGAGTACATGATCC − 3′, the reverse primer for CTSD was 5′- CGACACCTTGAGCGTGTAG − 3′, the forward primer for RAB5A was 5′- CAAGGCCGACCTAGCAAATAA − 3′, the reverse primer for RAB5A was 5′- GATGTTTTAGCGGATGTCTCCAT − 3′, the forward primer for GAPDH was 5′- GGAGCGAGATCCCTCCAAAAT − 3′, the reverse primer for GAPDH was 5′- GGCTGTTGTCATACTTCTCATGG − 3′, the forward primer for Itgα4 was 5′- GCTCTGGATTAGGGAGCAGTG − 3′, the reverse primer for Itgα4 was 5′- TACAGCTCTGTTGGGAATGCT − 3′, the forward primer for Itgα5 was 5′- GGCTTCAACTTAGACGCGGA − 3′, the reverse primer for Itgα5 was 5′- GGCCGGTAAAACTCCACTGA − 3′, the forward primer for ItgαV was 5′- AGGCACCCTCCTTCTGATCC − 3′, the reverse primer for ItgαV was 5′- GCGGGTAGAAGACCAGTCAC − 3′, the forward primer for Itgβ1 was 5′- CGCCGCGCGGAAAAGATG − 3′, the reverse primer for Itgβ1 was 5′- AAACACCAGCAGCCGTGTAA − 3′, the forward primer for Itgβ3 was 5′- ACCAGTAACCTGCGGATTGG − 3′, the reverse primer for was Itgβ3 5′- TCCGTGACACACTCTGCTTC − 3′, the forward primer for Itgβ4 was 5′- GCTCTGGATTAGGGAGCAGTG − 3′, the reverse primer for Itgβ4 was 5′- TACAGCTCTGTTGGGAATGCT − 3′, the forward primer for Itgβ5 was 5′- GGCTTCAACTTAGACGCGGA − 3′, the reverse primer for Itgβ5 was 5′- GGCCGGTAAAACTCCACTGA − 3′.

### Transwell assays and wound healing

For transwell migration, 1 × 10^5^/ml cells were suspended in 200 μL medium containing 0.1% bovine serum albumin into upper chamber of 24-well transwell plates (8 μm pore size; Corning), and 500 μL of medium containing 10%FBS was added to the lower chambers. After 24 h co-culture, the cells on the lower surface of membrane were fixed in 4% paraformaldehyde, stained by 0.1% crystal violet. The stained cells were then counted under Nikon light microscope (Nikon Corporation). Photographs of random fields across three replicate wells under 200 times magnification were captured for analysis.

For the wound-healing assay, monolayer cells were scratched with a sterile plastic tip with 95% confluence rate, washed with PBS three times, then cultured for 24 h with serum free medium. Photographs of random fields across three replicate wells were captured for analysis under Nikon light microscope (Nikon Corporation).

### Immunohistochemistry

TFEB were detected with use of PC tissue microarrays by Outdo Biotech containing 71 paired PC and peritumoral tissue samples and 28 PC (TMA; HPan-Ade170Sur-01, Shanghai, China). In brief, samples were embedded in paraffin and cut at a thickness of 4 μm. Sections and TMA were stained with hematoxylin and eosin (H&E) or incubated with primary antibodies (as indicated), using the ElivisionTM plus Polyer HRP IHC Kit (Maxim, Fujian, China). Images of representative fields were obtained from Aperio ImageScope. The overall score for each section was assessed by multiplying the intensity score by the percentage score of positively stained cells.

### Animal experiments

Animal experiments were approved by the Institutional Animal Care and Treatment Committee of Huazhong University of Science and Technology.

In vivo liver metastasis model: Female nude BALB/c mice obtained from HFK BioTechnology at 6 to 8 weeks old were injected with a stable TGF-β overexpression or simultaneous TGF-β overexpression and TFEB knockdown PC cells (2 × 10^6^ in 100 μL DMEM per mouse) into the spleens. Body weight of the mice was measured once a week from the time of implantation. Survival time of the mice was recorded until they were euthanized at 7 weeks after inoculation. And their liver tissues were harvested, imaged, embedded in 10% paraffin, and subjected to IHC staining.

*LSL-KrasG12D/Pdx1-Cre* mice model: Three-mouth-old *LSL-KrasG12D/Pdx1-Cre* mice (*n* = 6) were microinjected with recombinant mouse TGF-β (250 ng per mouse) according to the indicated frequency. The mice in the control group were intraperitoneally (i.p.) injected with vehicle (10% DMSO, 40% Cremophor/ethanol (3:1), and 50% PBS), while the mice in the alantolactone treatment group received an i.p. injection of alantolactone (3 mg/kg of body weight) simultaneously according to the indicated frequency. When the mice grow to 7 months old, they were euthanized and their pancreas tissues were harvested, imaged, embedded in 10% paraffin, and subjected to IHC staining for analysis.

### Expression profiling in TCGA dataset

TCGA PC mRNA gene expression data and relevant clinical information were downloaded from UCSC Xena at https://xenabrowser.net/ and cBioPortal for Cancer Genomics at http://www.cbioportal.org/. The gene expression profile was analyzed using the Illumina HiSeq pancan normalized pattern.

### Integrin internalization/recycling ELISA assay

Integrin internalization and recycling ELISA assays were performed as previously described [23]. The cells were biotinylated with cell surface integrin using Sulfo-NHS-SS biotin (0.2 mg/mL) in cold PBS. The cells were then incubated at 37 °C for a specified period of time while the control cells were kept on ice. Cell surface biotin was eluted with MesNa (50 mM) in PBS, followed by washing MesNa in the culture solution with iodoacetamide (20 mM) in PBS. The cells were further lysed to extract the protein and measured the concentration. The biotinylated protein was immunoprecipitated from an equal amount of total protein by streptavidin agarose beads. After beads washed with pre-cooled PBS, Itgα5 were detected by ELISA assay. In the integrin recycling assay, the previous biotin labeling step was consistent with the internalization experiment. The cells were internalized at 37 °C for 30 min, and the cell surface biotin was eluted with the above solution (MesNa, iodoacetamide). The cells were again placed at 37 °C and a second cell surface biotin elution was performed at the indicated times. Finally, the ELISA detection step in the internalization experiment was repeated to perform the detection of circulating integrin.

### Statistical analysis

Data are presented as the means ± SD from at least three separate experiments unless otherwise indicated. Significance was evaluated by a two-tailed Student’s t-test using GraphPad Prism 5 software (GraphPad Software Inc., La Jolla, CA, USA). *P* < 0.05 was considered statistically significant.

## Results

### TFEB is induced upon TGF-β treatment via the canonical smad pathway in Smad4-positive PC cells

**Fig. 1:**
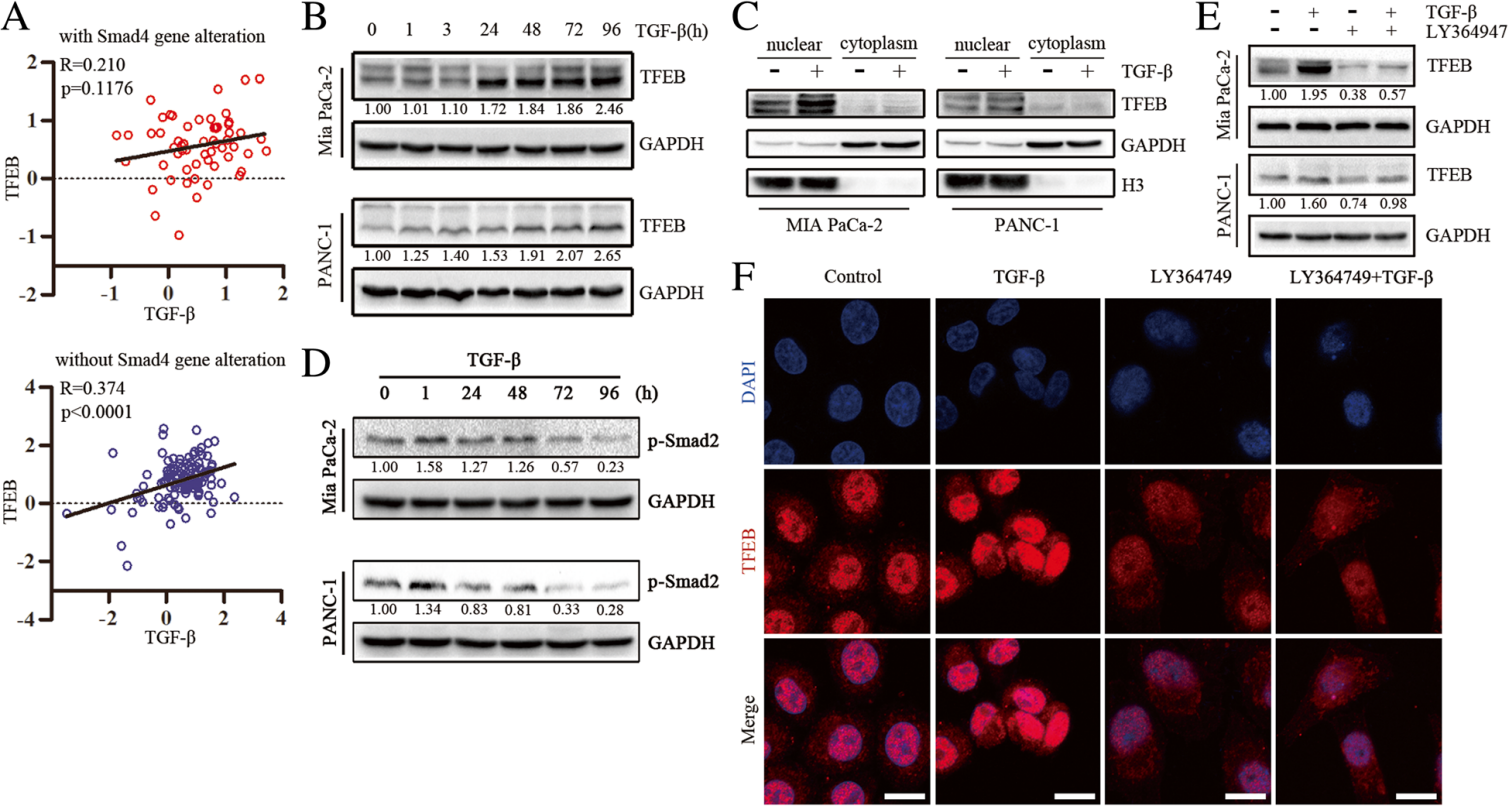
TFEB is induced upon TGF-β treatment via the canonical smad pathway in Smad4-positive PC cells. **a** Analysis of expression correlation based on a TCGA dataset of 184 pancreatic cancer patients with or without smad4 gene mutation or deletion. **b** Western blot revealing TFEB protein expression in MIA PaCa-2 and PANC-1 cells by treatment with TGF-β (10 ng/mL) for the times indicated. **c** Cell fractionation was performed to separate nuclear and cytoplasmic protein fraction from without or with TGF-β (10 ng/mL) for 48 h in MIA PaCa-2 and PANC-1 cells. **d** Western blot showing phosphorylated Smad2 (p-Smad2) expression in MIA PaCa-2 and PANC-1 cells by treatment with TGF-β (10 ng/mL) for the times indicated. **e** Western blot revealing TFEB expression in MIA PaCa-2 and PANC-1 cells treated with TGF-β (10 ng/mL) and/or LY364947 (10 μmol/L) for 48 h. **f** Immunofluorescence of PANC-1 cells after treated with TGF-β (10 ng/mL) and/or LY364947 (10 μmol/L) for 48 h

TFEB, which drives molecular clearance, is a master regulator of lysosomal biogenesis and autophagy [20, 21]. To examine whether the potential correlation between TGF-β and TFEB in PC, we analyzed a TCGA dataset including 184 PC patients with TGF-β and TFEB mRNA expressions [24, 25]. The expression of TGF-β mRNA was associated with TFEB mRNA expression, and there was a statistically significant difference between TGF-β and TFEB expression (Additional file 1: Figure S1A). Smad4 is a core mediator of the TGF-β signaling pathway that plays a pivotal role in the switch of TGF-β caused biological function [26]. We also found that TGF-β remained strongly correlated with TFEB in 126 cases without Smad4 gene alteration, but not in 58 cases with smad4 gene mutation or deletion (Fig. 1a). We then evaluated the expression of Smad4 in a panel of PC cell lines and found that AsPC-1, BxPC-3 and CFPAC-1 cell lines showed no Smad4 expression, while Smad4 expression was detectable in PANC-1, MIA PaCa-2 and Panc03.27 cell lines (Additional file 1: Figure S1B). Intriguingly, we confirmed that the level of TFEB increased in response to TGF-β in a time-dependent manner in Smad4-positive cell lines (MIA PaCa-2, PANC-1 and Panc03.27 lines), whereas TFEB was not induced by TGF-β in cell lines that lacked Smad4 expression (AsPC-1, BxPC-3 and CFPAC-1 lines) (Fig. 1b and Additional file 1: Figure S1C). We also performed western blot analysis of nuclear and cytoplasmic fractions and found that TGF-β treatment resulted in increased TFEB expression and nuclear translocation in Smad4-positive MIA PaCa-2 and PANC-1 cells (Fig. 1c). Consistent with the western blot data, treatment of PANC-1 cells with TGF-β increased the fluorescence staining of TFEB, particularly in the nucleus (Fig. 1f). Notably, down-regulation of Smad4 reduced TGF-β-induced TFEB, indicating a requirement for Smad4 in TGF-β-mediation induction of TFEB (Additional file 1: Figure S1D).

We next more closely examined the signaling pathways underlying TFEB induction by TGF-β. TGF-β treatment significantly induced smad2 phosphorylation (p-smad2) in MIA PaCa-2 and PANC-1 cells (Fig. 1d), but had little effect on levels of phosphorylated p44/42 MAP kinase and phosphorylated p38 MAP kinase (Additional file 1: Figure S1E). Co-IP assays demonstrated that TGF-β induced formation of the smad2/3/4 complex (Additional file 1: Figure S1F). Abundance of TFEB induced by TGF-β could be abrogated by the addition of the TβR1 inhibitor LY364947 (Fig. 1e and f). Taken together, these data indicated that TFEB expression was induced by TGF-β via the canonical smad pathway in Smad4-positive PC cells.

### TFEB is involved in TGF-β-induced autophagy in PC cells

**Fig. 2:**
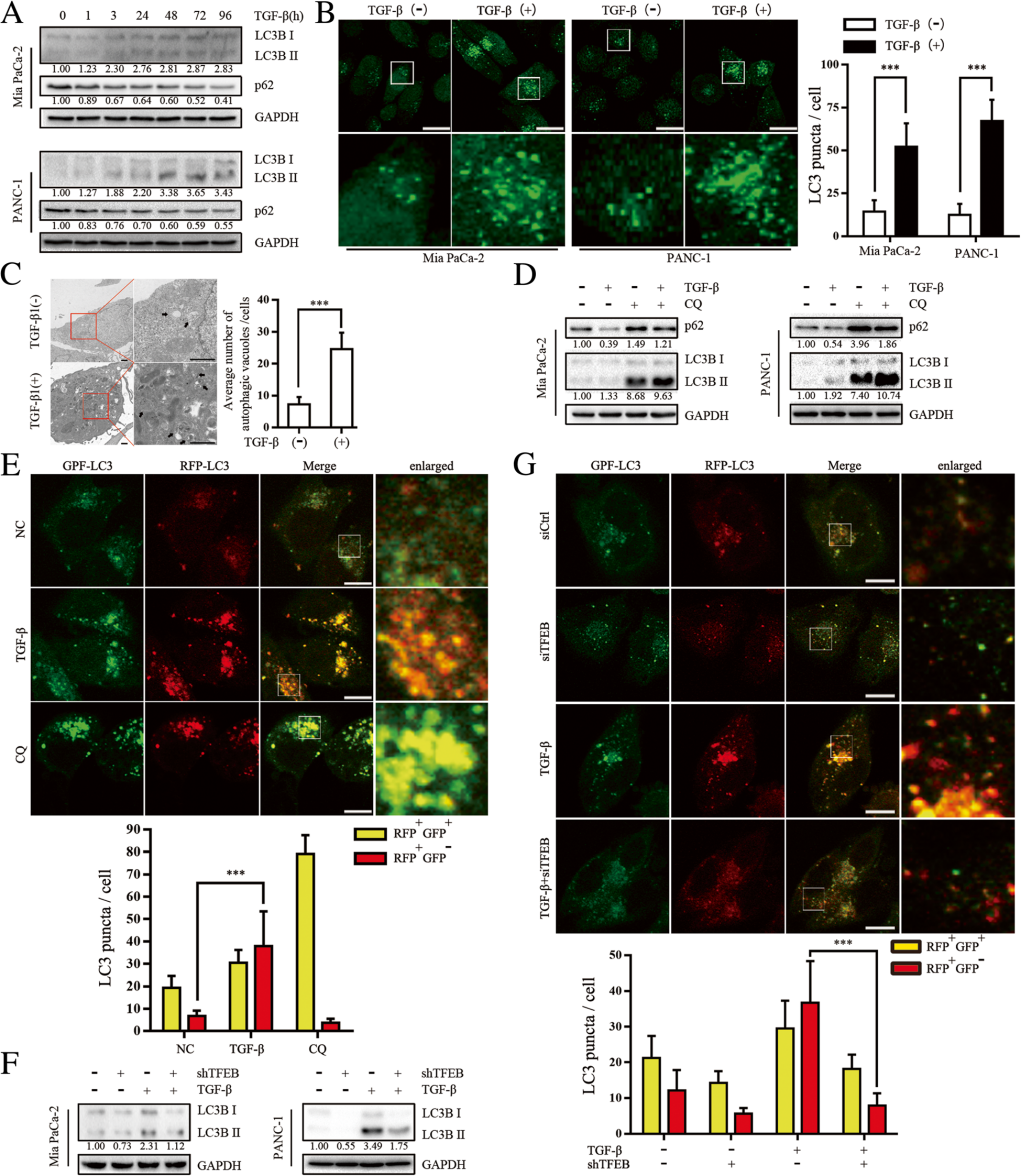
TFEB is involved in TGF-β-induced autophagy in PC cells. **a** Western blot showing expression of LC3B and p62 in MIA PaCa-2 and PANC-1 cells treated with TGF-β (10 ng/mL) for the times indicated. **b** MIA PaCa-2 and PANC-1 cells transfected with GFP-LC3B were treated with TGF-β (10 ng/mL) for 48 h, and representative confocal images are shown in the left panel; the number of LC3 dots is quantified in the right panel, and at least 30 cells were included in each group. ***, *p* < 0.001; Scale bar: 20 μm. **c** Transmission electron microscopy showing formation of autophagosomes/autolysosomes after TGF-β treatment (10 ng/mL, 48 h) in PANC-1 cells. Representative images and their magnified view are shown in the left panel. The right panel is the quantification of the number of autophagic vacuoles from at least 15 areas. Arrowhead: autophagic vacuoles; ***, *p* < 0.001; Scale bar: 1 μm. **d** Western blot revealing LC3B and p62 after TGF-β treatment (10 ng/mL, 24 h) in the absence or presence of CQ (10 μM, 24 h) in MIA PaCa-2 and PANC-1 cells. **e** PANC-1 cells transfected with GFP-mRFP-LC3B were treated with TGF-β (10 ng/mL, 48 h) or CQ (10 μM, 24 h) or (**g**) pre-transfected with shTFEB followed by a 48-h treatment with TGF-β (10 ng/mL). Representative confocal images of autophagosome (yellow puncta) and autolysosome (red puncta) formation are presented in the upper panel. Scale bar: 20 μm. The numbers of RFP^+^GFP^+^ LC3 puncta and RFP^+^GFP^−^ LC3 puncta are shown in the lower panel. ***, *p* < 0.001 compared with the control. **f** Western blot showing LC3B expression in MIA PaCa-2 and PANC-1 cells by transfected with shTFEB and/or treated with TGF-β (10 ng/mL, 48 h)

Western blot analysis of the autophagic marker microtubule-associated protein 1 light chain 3β (LC3B) demonstrated that LC3B II was significantly induced by TGF-β in a time-dependent manner in MIA PaCa-2 and PANC-1 cells. Interestingly, the abundance of p62 (SQSTM1), an LC3B binding protein that is degraded via autophagy, was markedly decreased in cells treated with TGF-β (Fig. 2a). However, no change in LC3B II was observed in AsPC-1, BxPC-3 and CFPAC-1 cells treated with TGF-β (Additional file 2: Figure S2A). Immunofluorescence verified the effect of TGF-β in inducing autophagy, as shown by an increased number of GFP-LC3B puncta in GFP-LC3B-transfected PC cells (Fig. 2b). Transmission electron microscopy revealed a two-fold increase in the number of autophagosomes/autolysosomes in response to chronic TGF-β treatment in PANC-1 cells (Fig. 2c). LC3B accumulation in cells may be due to either accelerated autophagosome synthesis or reduced autophagic vacuole maturation and degradation. To more closely examine how TGF-β induces autophagy, we evaluated the LC3B II and p62 levels in the absence and presence of CQ, which inhibits late-stage autophagy and the turnover of LC3B II (that is, autophagic flux) [12]. The induction of LC3B by TGF-β was confirmed through the use of CQ (Fig. 2d). We also used a tandem-labeled GFP-mRFP-LC3 reporter to measure autophagic flux. In this system, the GFP-mRFP-LC3 reporter localizes as yellow puncta in autophagosomes and red-only puncta in autolysosomes, as the GFP fluorescence decays in the acidic lysosomal environment, whereas mRFP is more resistant to low pH [27]. As shown in Fig. 2e, TGF-β increased the number of red-only LC3 puncta in PANC-1 cells, indicating an increase of autophagic flux (Additional file 2: Figure 2E).

To ascertain whether TFEB plays a significant role in TGF-β-induced autophagy, we performed gene silencing using shRNA targeting TFEB (Additional file 2: Figure S2B). Down-regulation of TFEB not only reduced the induction of LC3B in response to TGF-β in MIA PaCa-2 and PANC-1 cells compared with TGF-β treatment alone (Fig. 2f), but also decreased the number of red-only LC3 puncta in GFP-mRFP-LC3-transfected PANC-1 cells compared with cells treated with TGF-β (Fig. 2g). Interestingly, knockdown of ATG5 (an essential autophagy gene) attenuated TGF-β caused accumulation of autophagosomes, but did not alter the expression of TFEB (Additional file 2: Figure S2C). Collectively, these results demonstrated that TGF-β induces autophagy during the early stage in PC cells and that TGF-β-regulated autophagy is dependent on TFEB.

### TFEB-driven autophagy is required for TGF-β-induced migration and metastasis of PC cells

**Fig. 3:**
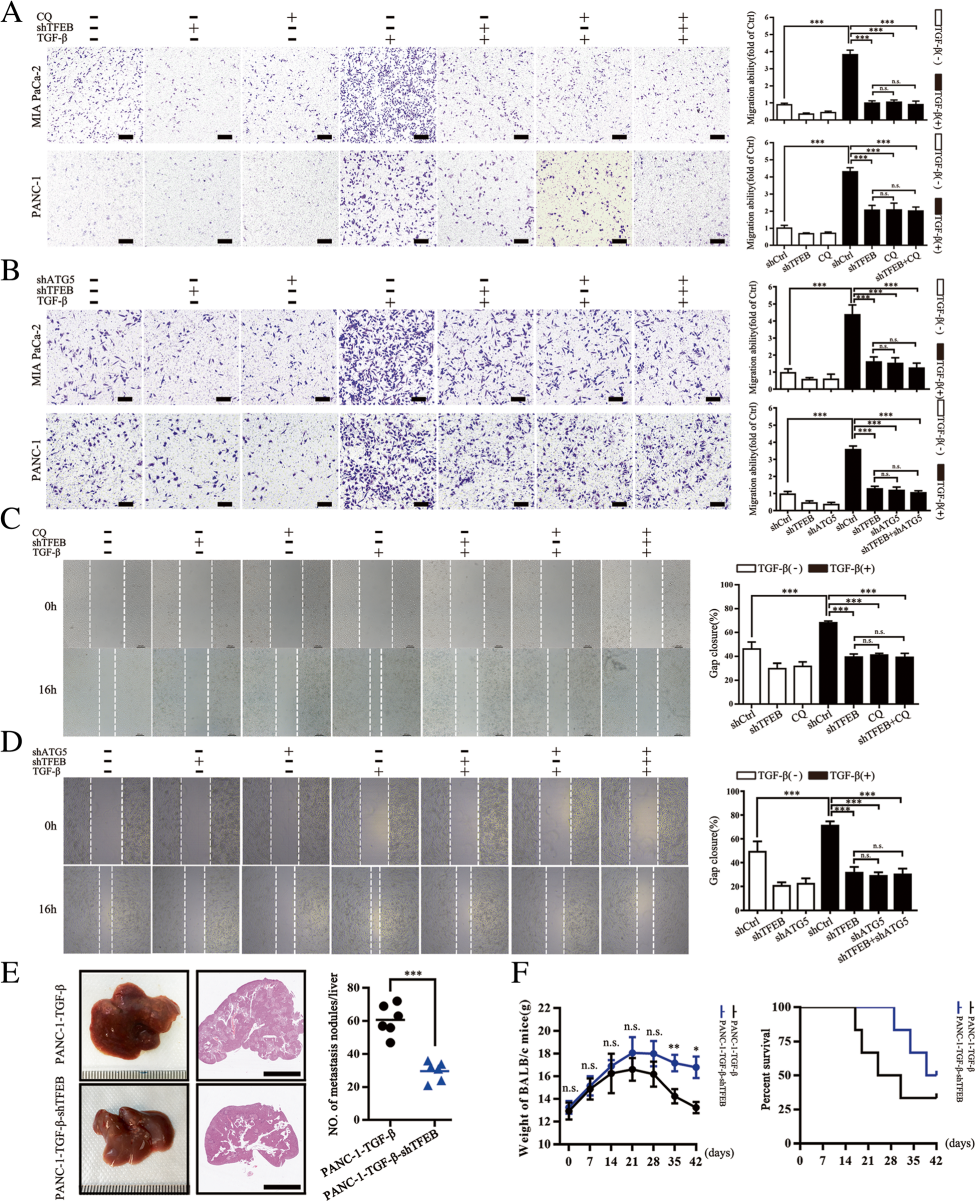
TFEB-driven autophagy is required for TGF-β-induced migration and metastasis of PC cells. **a** Transwell assay showing migrated MIA PaCa-2 and PANC-1 cells pre-transfected with shTFEB or/and CQ followed by a 48-h treatment with TGF-β (10 ng/mL). Data are presented as the means ± SD from 3 independent experiments (***, p < 0.001). **b** Transwell assay showing migrated MIA PaCa-2 and PANC-1 cells transfected with shTFEB or/and shATG5 followed by a 48-h treatment with TGF-β (10 ng/mL). Data are presented as the means ± SD from 3 independent experiments (***, *p* < 0.001). **c** Wound healing assays were used to examine cell migration in PANC-1 cells pre-transfected with shTFEB or/and CQ followed by a 48-h treatment with TGF-β (10 ng/mL); the right panel is the quantification of the wound-healing percentages. **d** Wound healing assays were used to examine cell migration in PANC-1 cells transfected with shTFEB or/and shATG5 followed by a 48-h treatment with TGF-β (10 ng/mL); the right panel is the quantification of the wound-healing percentages. **e** Liver metastasis model by nude BALB/c mice, the left panel is representative image, statistical analysis of the average numbers of visible liver is in right panel. Data are presented as mean ± SD (*n* = 6). Scale bar: 5 mm. ***, *p* < 0.001. **f** The body weight of the mice in each group was measured once a week from the time of cells injection and Kaplan-Meier survival curves of each indicated group (*n* = 6). n.s., no significance; *, *p* < 0.05; **, *p* < 0.01

Given the role of TGF-β in stimulating TFEB-regulated autophagy in PC, we next examined the potential function of TFEB in TGF-β involving tumor biological behavior. TFEB knockdown significantly decreased TGF-β-induced migration ability of MIA PaCa-2 and PANC-1 cells, and CQ treatment or ATG5 knockdown showed similar effects (Fig. 3a-d). Simultaneous TFEB knockdown and autophagy blockade (CQ treatment or down-regulation of ATG5) did not further inhibit TGF-β-induced motility, suggesting that TFEB-driven autophagy was responsible for TGF-β-induced migration. We next examined the physiological relevance of TFEB in in vivo mouse models. In the liver metastasis model, TFEB knockdown reduced liver metastases, prevented weight loss and improved survival of mice (Fig. 3e and f). In the *LSL-KrasG12D/Pdx1-Cre* mice model, alantolactone, a TFEB inhibitor [28], was used to examine whether pharmacological down-regulation of TFEB affected PC progression or characteristics (Additional file 3: Figure S3A). As expected, when mice grew 7 months of age, much of the normal pancreatic architecture was replaced by abnormal structure (PanIN or PDAC) in *LSL-KrasG12D/Pdx1-Cre* mice with recombinant mouse TGF-β injection and alantolactone weakened the lesions (Additional file 3: Figure S3B). Overall, these results revealed that TFEB-driven autophagy plays an important role in TGF-β-induced migration and metastasis of PC. These findings also suggest that TFEB inhibition decelerates the progression to more advanced stages of PC.

### TGF-β facilitates endocytosis of integrin and focal adhesion disassembly mediated by TFEB

**Fig. 4:**
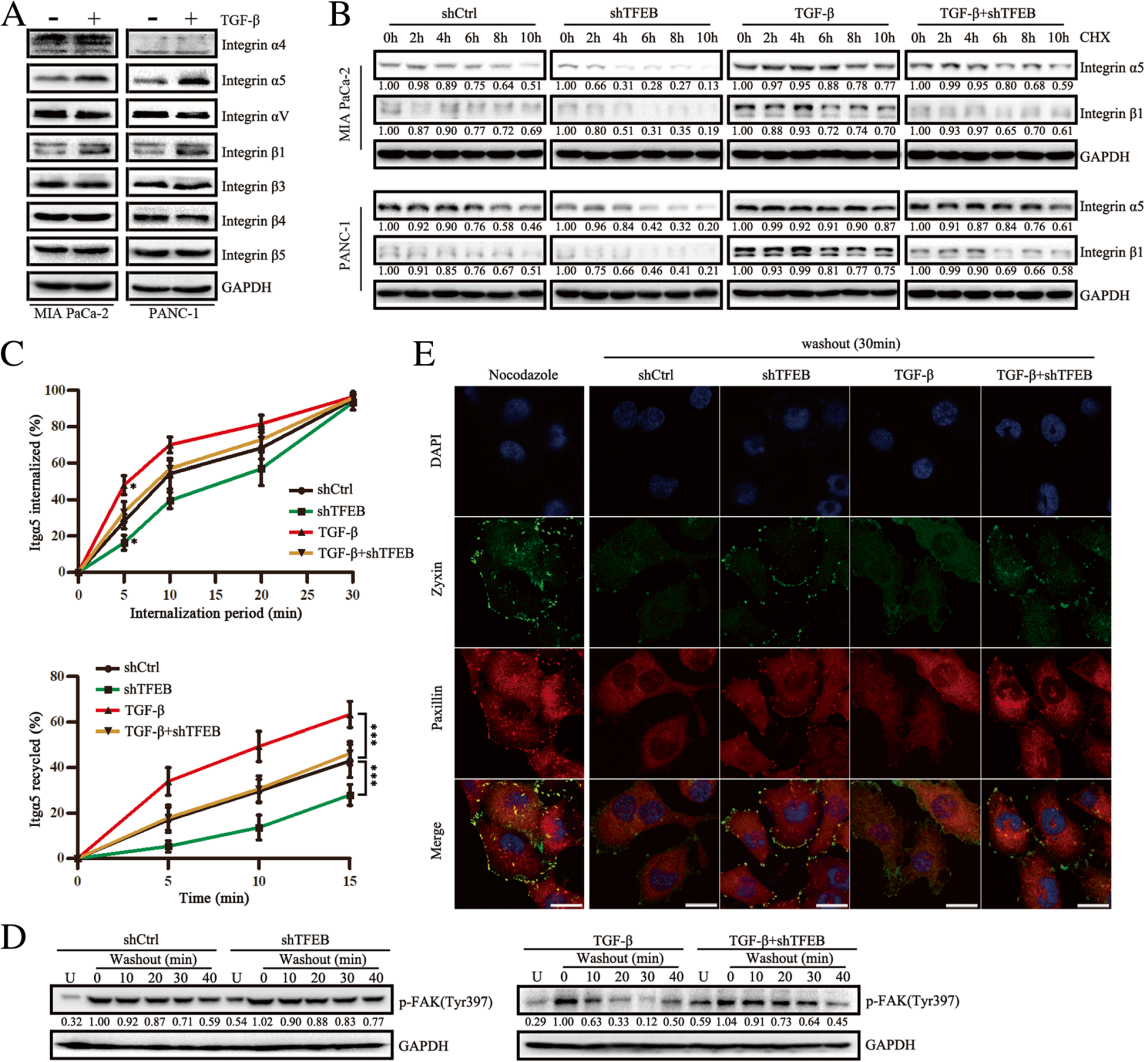
TGF-β facilitates endocytosis of integrin and focal adhesion disassembly mediated by TFEB. **a** Western blot revealing expression of indicated proteins in MIA PaCa-2 and PANC-1 cells after treatment with TGF-β (10 ng/mL, 48 h). **b** Itgα5β1 levels in MIA PaCa-2 and PANC-1 cells treated with CHX (25μg/mL) over the indicated periods after pre-transfected with shTFEB followed by a 48-h treatment with TGF-β (10 ng/mL). **c** Biotinylation-based internalization and recycling assays showing internalized and recycled Itgα5 in PANC-1 cells after pre-transfected with shTFEB followed by a 48-h treatment with TGF-β (10 ng/mL). **d** Western blot of focal adhesion disassembly assay via assessing p-FAK (Tyr397) expression after nocodazole washout (U means untreated). **e** Confocal images of paxillin and zyxin in PANC-1 cells pre-transfected with shTFEB followed by a 48-h treatment with TGF-β (10 ng/mL)

Endocytosis of integrin and its regulated focal adhesion disassembly play well-documented roles in cell motility involving endosomal pathway [29, 30]. To evaluate whether endocytosis of integrin participates in TFEB-driven autophagy regulating TGF-β-induced cell migration, we first performed bioinformatics analysis of the expression of TGF-β and various integrin in the 184 PC patients [25]. The expression of TGF-β was associated with integrin, particularly Itgα5 (Additional file 4: Figure S4A). However, we found that TGF-β had no effect on the transcriptional expression of the integrin (Additional file 4: Figure S4B). We further found that TGF-β treatment increased Itgα5β1 protein levels in MIA PaCa-2 and PANC-1 cells and led to a noticeable increase in the half-lives of Itgα5 and Itgβ1 in CHX chase assays (Fig. 4a and b). TFEB knockdown not only shortened the half-life of Itgα5β1 protein, but also abrogated the increase of it treated with TGF-β, indicating TFEB was responsible for lengthening half-lives of Itgα5β1 induced by TGF-β (Fig. 4b). Biotinylation-based internalization and recycling assays revealed that Itgα5 was internalized more rapidly in TGF-β treatment cells, and the return to the cell surface was severely promoted (Fig. 4c). TFEB knockdown not only reduces internalization and recycling of Itgα5, but abrogated the effect of them treated with TGF-β (Fig. 4c). We next performed focal adhesion disassembly assays using nocodazole. Treatment of cells with nocodazole, a microtubule-disrupting agent, inhibits focal adhesion disassembly, and after washout, the loss of focal adhesions is correlated with decreased p-FAK (Tyr397); the more rapid loss of p-FAK (Tyr397) reflects more rapid focal adhesion disassembly [31]. Treatment of cells with TGF-β accelerated the loss of p-FAK (Tyr397) compared with controls; however, TFEB knockdown decelerated the loss of p-FAK (Tyr397) caused by TGF-β (Fig. 4d). Consistent with these results, immunofluorescence for focal adhesion proteins paxillin and zyxin demonstrated there were fewer focal adhesions in TGF-β-treated PANC-1 cells at 30 min after nocodazole washout, while there were much more focal adhesions in TFEB knockdown PANC-1 cells (Fig. 4e). Thus, these results revealed that TFEB plays an important role in the regulation of TGF-β-mediated endocytosis of Igtα5β1 and focal adhesion disassembly.

### RAB5A is TFEB direct target for TGF-β induced endocytosis of integrin

**Fig. 5:**
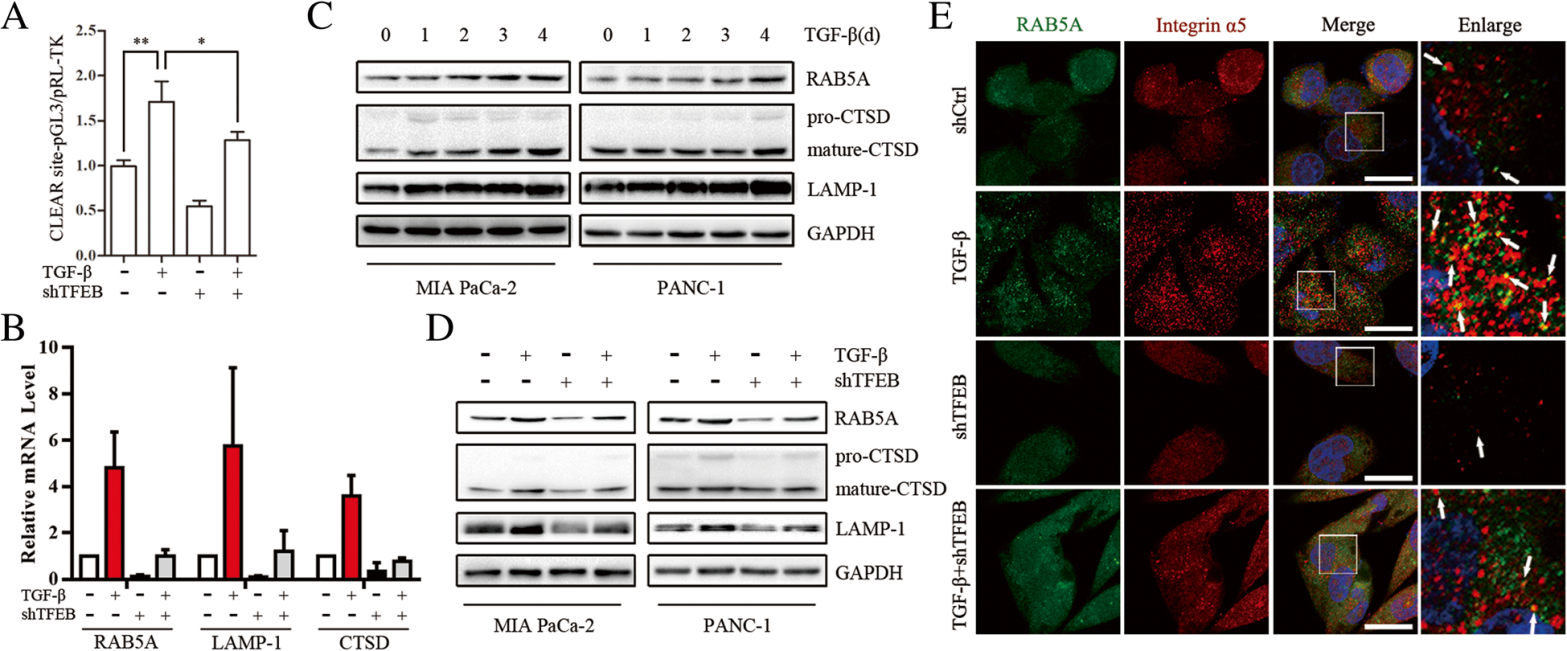
RAB5A is TFEB direct target for TGF-β induced endocytosis of integrin. **a** PANC-1 cells were co-transfected with pRL-TK and CLEAR site-pGL3 and treated with TGF-β (10 ng/mL, 48 h) and/or pre-transfected with shTFEB. Luciferase activity was normalized to renilla luciferase activity and expressed as the mean ± SD. *, *p* < 0.05; **, *p* < 0.01. **b** Real-time PCR showing relative mRNA level of RAB5A, LAMP-1 and CTSD in PANC-1 cells treated with TGF-β (10 ng/mL, 48 h) and/or pre-transfected with shTFEB. **c** Western blot analysis of RAB5A, LAMP-1 and CTSD in MIA PaCa-2 and PANC-1 cells by treatment with TGF-β (10 ng/mL) for the times indicated. **d** Western blot revealing relative mRNA level of RAB5A, LAMP-1 and CTSD in PANC-1 cells treated with TGF-β (10 ng/mL, 48 h) and/or pre-transfected with shTFEB. **e** Confocal images of RAB5A and Itgα5 in PANC-1 cells pre-transfected with shTFEB followed by a 48-h treatment with TGF-β (10 ng/mL)

TFEB is a master regulator of endosomal/lysosomal biogenesis and function and regulates the CLEAR gene network [20]. We performed luciferase reporter assays to examine whether CLEAR gene network was controlled by TGF-β. Activity of the luciferase construct was elevated in the presence of TGF-β in PANC-1 cells and could be reversed by simultaneous down-regulation of TFEB (Fig. 5a). We evaluated the promoter sequences of three known endosomal/lysosomal function-related genes (RAB5A, LAMP-1 and CTSD genes) and found a strong enrichment for the TCACGTG motif consensus sequence (Additional file 4: Figure S4C), indicating that TFEB may transcriptionally target these proteins in PC cells. We also confirmed that RAB5A, LAMP-1 and CTSD mRNA and protein levels were up-regulated in TGF-β-treated PC cells (Fig. 5b-d). However, TFEB knockdown abrogated the transcriptional increase of RAB5A, LAMP-1 and CTSD mRNAs, revealing that RAB5A is a direct target of TFEB in PC cells (Fig. 5b and d).

RAB5A, a master regulator of endocytosis, is sufficient to induce collective cellular motility [32]. To examine whether RAB5A is involved in TGF-β-induced endocytosis of integrin, we performed immunofluorescence of RAB5A and Igtα5. Confocal microscopy demonstrated that RAB5A colocalized with Igtα5 puncta in TGF-β-treated PANC-1 cells, while TFEB knockdown decreased the colocalization of RAB5A and Igtα5 (Fig. 5e). These results demonstrate that RAB5A is a direct target of TFEB controlled by the CLEAR network, and RAB5A plays a critical role in TGF-β-induced endocytosis of integrin.

### Aberrant expression of TFEB and RAB5A in PC

**Fig. 6:**
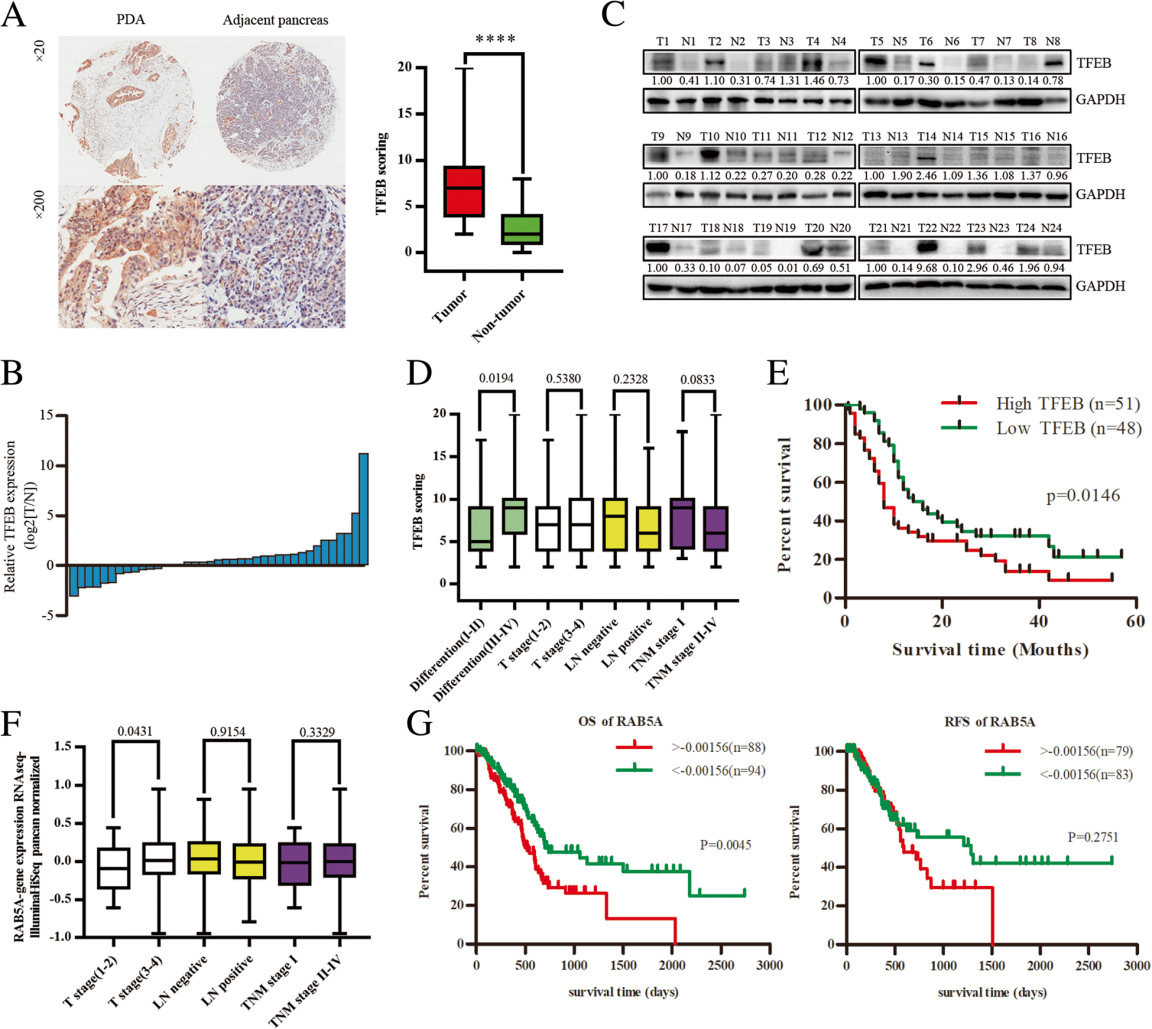
Aberrant expression of TFEB and RAB5A in PC. **a** Immunohistochemical analysis of TFEB expression in PC tissues. Representative images are shown in the left panel; statistical analysis of TFEB expression is in right panel. ****, *p* < 0.0001. **b** Real-time PCR showing relative mRNA level of TFEB in 39 paired PC tissues. **c** Western blot revealing protein TFEB expression in 39 paired PC tissues. **d** Relative expression scores of TFEB in PC with or without poor differentiation, T stage (1–2), positive lymphatic metastasis and TNM stage I were showed as box plots. **e** Kaplan-Meier analysis of the correlation between TFEB expression and overall survival of PC patients. **f** Relative expression of RAB5A in PC with or without T stage (1–2), positive lymphatic metastasis and TNM stage I were showed as box plots. **g** Kaplan-Meier analysis of the correlation between relative expression of RAB5A and overall survival or relapse-free survival of PC patients

We next examined the clinical significance of TFEB by evaluating its expression in a PC tissue microarray and the correlation with clinicopathological features (Additional file 5: Figure S5A). Immunohistochemistry assays showed that TFEB expression level was significantly enhanced in PC tissues compared with adjacent tissues (Fig. 6a). Real-time PCR in 39 cases with paired PC and adjacent tissues revealed that TFEB mRNA level was much higher in PC tissues (Fig. 6b). Western blot also showed TFEB up-regulation in an additional 24 paired PC samples and PC cell lines (Fig. 6c and Additional file 5: Figure S5B). Clinicopathological analysis of TFEB expression revealed that elevated expression of TFEB was significantly associated with poorly differentiated tumors (Fig. 6d). Kaplan-Meier analysis showed that patients with elevated expression of TFEB had a shorter overall survival; the median survival of PC patients with high TFEB expression was 8 months, which was significantly shorter than that of patients with low TFEB expression (16 months) (Fig. 6e).

Given that RAB5A was direct target of TFEB controlled by the CLEAR network in TGF-β-induced endocytosis of integrin, we hypothesized that high RAB5A expression may be associated with poor survival. Indeed, analysis of data from the 184 PC patients in the TCGA dataset revealed that enhanced RAB5A mRNA level was significantly associated with advanced tumor stage (Fig. 6f). Kaplan-Meier analysis showed that patients with higher expression of RAB5A had a shorter overall survival than patients with lower RAB5A expression (Fig. 6g). However, no statistical difference was observed in relapse-free survival time in the RAB5A high expression and low expression groups (Fig. 6h). Taken together, these data indicated that the expression of TFEB is elevated in PC. Furthermore, enhanced TFEB expression and its direct target RAB5A both predict poor prognosis in PC patients and may contribute to the progression of PC.

## Discussion

PC remains one of the most aggressive cancers. The complicated tumor microenvironment, particularly TGF-β, a multifunctional cytokine that regulates multiple critical biological processes, provides possible convenience for the progression of PC cells [3, 6, 33]. Here, we demonstrated that TGF-β induces TFEB expression via the canonical smad pathway in Smad4-positive PC cells and facilitates TFEB-mediated autophagic activation. We provide evidence that TFEB-driven autophagy is required for TGF-β-induced migration and metastasis of PC cells, because autophagy modulating effects of TFEB is in charge of endocytosis of Itgα5β1 and focal adhesion disassembly through RAB5A transcriptional activation controlled by the CLEAR network in PC cells. We further showed that enhanced expression of TFEB and its direct target RAB5A both predict poor prognosis in PC patients. This study suggests that TFEB-driven autophagy contributes to the progression of PC. Increasing TFEB expression in PC cells may be an intrinsic feature of the TGF-β-induced migration and metastasis. These results indicate that TFEB may be a potential target for cancer therapy.

PC cells are often exposed to microenvironment with increased TGF-β via a kind of paracrine signaling, which promotes the microenvironment to support cancer progression [34]. Although reduced TFEB expression was identified driving by secreted TGF-β in tumor-associated macrophages [35], we showed that treatment of TGF-β up-regulates TFEB in Smad4-positive PC cells. Previous studies also revealed that starvation inhibits Erk-mediated TFEB phosphorylation and induces TFEB translocation from the cytoplasm to the nucleus [21, 35, 36]. However, our data indicate that activation of TFEB by TGF-β does not occur through non-canonical smad signaling involving Erk and p38 MAPK, but rather in a Smad4-dependent manner.

Like many other tumor types, PC exhibits elevated levels of autophagy compared with normal tissue [13, 22]. Autophagy is a dynamic and continuous process that relies on the cooperation of two organelles: the autophagosome and the lysosome [12, 37]. TFEB regulates the expression of genes encoding enzymes involved in sequential steps of autophagic processes such as vesicle (endosome/lysosome) formation and lysosomal enzyme synthesis, among others [20, 21]. Previous studies also showed that TFEB induces expression of a number of core endocytic genes, including the RAB5 gene, and promotes endocytosis during starvation to sustain lysosomal function and autophagy [38]. We found that TFEB is responsible for TGF-β-induced autophagy and that TFEB-driven autophagy induced by TGF-β regulates RAB5A-dependent endocytosis of Itgα5β1 in PC cells. Our study provides a novel mechanism by which endocytosis of integrin is regulated by the TGF-β/TFEB/RAB5A signaling pathways in PC cells.

Previous studies showed that high expression of TGF-β correlates with more advanced stages of malignancy and decreased survival [39]. We demonstrated that TFEB-driven autophagy plays a crucial role in TGF-β-induced migration and metastasis of PC cells. Integrin endocytosis is an important event that regulates the invasion ability of cancer cells [30]. RAB5 is required for microtubule-dependent adhesion disassembly, and suppressing RAB5-dependent integrin endocytosis inhibits migration and invasiveness in tumor cells [29, 40, 41]. We showed that RAB5A is direct target of TFEB controlled by the CLEAR network, and RAB5A is critical in TGF-β-induced endocytosis of integrin in PC. Furthermore, both elevated TFEB expression and its direct target RAB5A predicted poor prognosis in PC patients and may contribute to the progression of PC. Together, our work demonstrates a critical role for TFEB-driven autophagy in endocytosis of Itgα5β1 and focal adhesion disassembly through transcriptional activation of RAB5A. These studies highlight the potential utility of suppressing TFEB-driven autophagy to block PC metastasis.

## Conclusion

Here we identified TFEB as a key regulator in TGF-β-induced autophagy in PC cells. TFEB-driven autophagy is required for TGF-β-induced migration and metastasis of PC cells via promoting endocytosis of Itgα5β1 and focal adhesion disassembly through the TGF-β-TFEB-RAB5A axis.

## Additional files

Additional file 1: **Figure S1A.** Analysis of expression correlation based on a TCGA dataset of 184 pancreatic cancer patients. **Figure S1B.** Western blot showing Smad4 levels in indicated PC cell lines. **Figure S1C.** Western blot revealing TFEB protein expression in Panc03.27, AsPC-1, BxPC-3 and CFPAC-1 cells by treatment with TGF-β (10 ng/mL) for the times indicated. **Figure S1D.** Western blot revealing TFEB and Smad4 by treatment with TGF-β (10 ng/mL, 48hous) or/and pretreated with siSmad4 in MIA PaCa-2 and PANC-1 cells. **Figure S1E.** Western blot showing phosphorylated p44/42 MAP kinase (p-Erk) and phosphorylated p38 MAP kinase (p-p38) expression in MIA PaCa-2 and PANC-1 cells by treatment with TGF-β (10 ng/mL) for the times indicated. **Figure S1F.** Co-immunoprecipitation analysis of Smad2/3-Smad4 heteromeric complex formation treated with TGF-β (10 ng/mL, 1 h) in PANC-1 cells. (TIF 1532 kb)

Additional file 2: **Figure S2A.** Western blot showing LC3B expression in AsPC-1, BxPC-3 and CFPAC-1 cells by treatment with TGF-β (10 ng/mL) for the times indicated. **Figure S2B.** Western blot revealing the efficiency of shRNA targeting TFEB in MIA PaCa-2 and PANC-1 cells. **Figure S2C.** Western blot showing LC3B and TFEB in MIA PaCa-2 and PANC-1 cells by transfected with shATG5 and/or treated with TGF-β (10 ng/mL, 48 h). (TIF 1039 kb)

Additional file 3: **Figure S3A.** The schematic illustration shows injection of TGF-β with or without alantolactone in *LSL-Kras*^*G12D*^*/Pdx1-Cre* mice model. Arrowhead: injection frequency. **Figure S3B.** Pancreatic pathology images reveal representative 7-month-old *LSL-Kras*^*G12D*^*/Pdx1-Cre* mice by treatment of TGF-β with or without alantolactone. Quantification of the percentage of normal ducts and PanIN or PDAC is shown in the right panel. (TIF 1274 kb)

Additional file 4: **Figure S4A.** Analysis of expression correlation as indicated based on a TCGA dataset of 184 pancreatic cancer patients. **Figure S4B.** Real-time PCR showing relative mRNA level of indicated integrin in PC cells. **Figure S4C.** TCACGTG motif analysis in promoter sequence of RAB5A, LAMP-1 and CTSD gene in *Homo sapiens*. (TIF 1313 kb)

Additional file 5: **Figure S5A.** Immunohistochemical analysis shows image of TFEB expression in PC tissues. **Figure S5B.** Western blot showing TFEB levels in indicated PC cell lines. (TIF 1090 kb)

## Data Availability

Please contact authors for data requests.

## Abbreviations

Alan: Alantolactone
CHX: Cycloheximide
CLEAR: Coordinated lysosomal expression and regulation
Co-IP: Co-Immunoprecipitation
CQ: Chloroquine
CTSD: Cathepsin D
HPDE: Human pancreatic duct epithelial cells
Itgα5: Integrin α5
Itgβ1: Integrinβ1
LAMP1: Lysosomal associated membrane protein 1
LC3B: Microtubule-associated protein 1 light chain 3β
PanIN: Pancreatic intraepithelial neoplasia
PC: Pancreatic cancer
PDAC: Pancreatic ductal adenocarcinoma
TFEB: Transcription factor EB
TGF-β: Transforming growth factor-β
TβR1: Transforming growth factor-β type I receptor

## References

[1] Bray Freddie, Ferlay Jacques, Soerjomataram Isabelle, Siegel Rebecca L., Torre Lindsey A., Jemal Ahmedin (2018). Global cancer statistics 2018: GLOBOCAN estimates of incidence and mortality worldwide for 36 cancers in 185 countries. CA: A Cancer Journal for Clinicians.

[2] Aoyagi Y, Oda T, Kinoshita T, Nakahashi C, Hasebe T, Ohkohchi N (2004). Overexpression of TGF-beta by infiltrated granulocytes correlates with the expression of collagen mRNA in pancreatic cancer. Br J Cancer.

[3] Kang Y, Roife D, Lee Y, Lv H, Suzuki R, Ling J (2016). Transforming growth factor-beta limits secretion of Lumican by activated stellate cells within primary pancreatic adenocarcinoma tumors. Clin Cancer Res.

[4] Lohr M, Schmidt C, Ringel J, Kluth M, Muller P, Nizze H (2001). Transforming growth factor-beta1 induces desmoplasia in an experimental model of human pancreatic carcinoma. Cancer Res.

[5] Pan B, Liao Q, Niu Z, Zhou L, Zhao Y (2015). Cancer-associated fibroblasts in pancreatic adenocarcinoma. Future Oncol.

[6] Biffi Giulia, Oni Tobiloba E., Spielman Benjamin, Hao Yuan, Elyada Ela, Park Youngkyu, Preall Jonathan, Tuveson David A. (2018). IL1-Induced JAK/STAT Signaling Is Antagonized by TGFβ to Shape CAF Heterogeneity in Pancreatic Ductal Adenocarcinoma. Cancer Discovery.

[7] Adorno M, Cordenonsi M, Montagner M, Dupont S, Wong C, Hann B (2009). A mutant-p53/Smad complex opposes p63 to empower TGFbeta-induced metastasis. Cell.

[8] Ellenrieder V, Hendler SF, Boeck W, Seufferlein T, Menke A, Ruhland C (2001). Transforming growth factor beta1 treatment leads to an epithelial-mesenchymal transdifferentiation of pancreatic cancer cells requiring extracellular signal-regulated kinase 2 activation. Cancer Res.

[9] Michl P, Ramjaun AR, Pardo OE, Warne PH, Wagner M, Poulsom R (2005). CUTL1 is a target of TGF (beta) signaling that enhances cancer cell motility and invasiveness. Cancer Cell.

[10] Zhao S, Venkatasubbarao K, Lazor JW, Sperry J, Jin C, Cao L (2008). Inhibition of STAT3 Tyr705 phosphorylation by Smad4 suppresses transforming growth factor beta-mediated invasion and metastasis in pancreatic cancer cells. Cancer Res.

[11] Fitzwalter BE, Thorburn A (2015). Recent insights into cell death and autophagy. FEBS J.

[12] Klionsky DJ, Abdelmohsen K, Abe A, Abedin MJ, Abeliovich H, Acevedo Arozena A (2016). Guidelines for the use and interpretation of assays for monitoring autophagy (3rd edition). Autophagy.

[13] Kimmelman AC, White E (2017). Autophagy and tumor metabolism. Cell Metab.

[14] White E (2015). The role for autophagy in cancer. J Clin Invest.

[15] Sharifi MN, Mowers EE, Drake LE, Collier C, Chen H, Zamora M (2016). Autophagy promotes focal adhesion disassembly and cell motility of metastatic tumor cells through the direct interaction of Paxillin with LC3. Cell Rep.

[16] Mowers EE, Sharifi MN, Macleod KF (2017). Autophagy in cancer metastasis. Oncogene.

[17] Kiyono K, Suzuki HI, Matsuyama H, Morishita Y, Komuro A, Kano MR (2009). Autophagy is activated by TGF-beta and potentiates TGF-beta-mediated growth inhibition in human hepatocellular carcinoma cells. Cancer Res.

[18] Suzuki HI, Kiyono K, Miyazono K (2010). Regulation of autophagy by transforming growth factor-beta (TGF-beta) signaling. Autophagy.

[19] Jiang Y, Woosley AN, Sivalingam N, Natarajan S, Howe PH (2016). Cathepsin-B-mediated cleavage of Disabled-2 regulates TGF-beta-induced autophagy. Nat Cell Biol.

[20] Palmieri M, Impey S, Kang H, di Ronza A, Pelz C, Sardiello M (2011). Characterization of the CLEAR network reveals an integrated control of cellular clearance pathways. Hum Mol Genet.

[21] Settembre C, Di Malta C, Polito VA, Garcia Arencibia M, Vetrini F, Erdin S (2011). TFEB links autophagy to lysosomal biogenesis. Science.

[22] Yang S, Wang X, Contino G, Liesa M, Sahin E, Ying H (2011). Pancreatic cancers require autophagy for tumor growth. Genes Dev.

[23] Dozynkiewicz MA, Jamieson NB, Macpherson I, Grindlay J, van den Berghe PV, von Thun A (2012). Rab25 and CLIC3 collaborate to promote integrin recycling from late endosomes/lysosomes and drive cancer progression. Dev Cell.

[24] Pancreatic Adenocarcinoma (TCGA, PanCancer Atlas) dataset. cBioPortal for Cancer Genomics, USA. http://www.cbioportal.org/. Accessed 4 May 2018.

[25] Pancreatic Cancer (PAAD) dataset. UCSC Xena, USA. https://xenabrowser.net/. Accessed 4 May 2018.

[26] Hezel AF, Kimmelman AC, Stanger BZ, Bardeesy N, Depinho RA (2006). Genetics and biology of pancreatic ductal adenocarcinoma. Genes Dev.

[27] Li X, Zhu F, Jiang J, Sun C, Zhong Q, Shen M (2016). Simultaneous inhibition of the ubiquitin-proteasome system and autophagy enhances apoptosis induced by ER stress aggravators in human pancreatic cancer cells. Autophagy.

[28] He R, Shi X, Zhou M, Zhao Y, Pan S, Zhao C (2018). Alantolactone induces apoptosis and improves chemosensitivity of pancreatic cancer cells by impairment of autophagy-lysosome pathway via targeting TFEB. Toxicol Appl Pharmacol.

[29] Chao WT, Kunz J (2009). Focal adhesion disassembly requires clathrin-dependent endocytosis of integrins. FEBS Lett.

[30] Paul NR, Jacquemet G, Caswell PT (2015). Endocytic trafficking of Integrins in cell migration. Curr Biol.

[31] Ezratty EJ, Partridge MA, Gundersen GG (2005). Microtubule-induced focal adhesion disassembly is mediated by dynamin and focal adhesion kinase. Nat Cell Biol.

[32] Malinverno C, Corallino S, Giavazzi F, Bergert M, Li Q, Leoni M (2017). Endocytic reawakening of motility in jammed epithelia. Nat Mater.

[33] Kubiczkova L, Sedlarikova L, Hajek R, Sevcikova S (2012). TGF-beta - an excellent servant but a bad master. J Transl Med.

[34] Biswas S, Criswell TL, Wang SE, Arteaga CL (2006). Inhibition of transforming growth factor-beta signaling in human cancer: targeting a tumor suppressor network as a therapeutic strategy. Clin Cancer Res.

[35] Fang L, Hodge J, Saaoud F, Wang J, Iwanowycz S, Wang Y (2017). Transcriptional factor EB regulates macrophage polarization in the tumor microenvironment. Oncoimmunology.

[36] Kim SH, Kim G, Han DH, Lee M, Kim I, Kim B (2017). Ezetimibe ameliorates steatohepatitis via AMP activated protein kinase-TFEB-mediated activation of autophagy and NLRP3 inflammasome inhibition. Autophagy.

[37] Settembre C, Ballabio A (2011). TFEB regulates autophagy: an integrated coordination of cellular degradation and recycling processes. Autophagy.

[38] Nnah Israel C., Wang Biao, Saqcena Chaitali, Weber Gregory F., Bonder Edward M., Bagley Dustin, De Cegli Rossella, Napolitano Gennaro, Medina Diego L., Ballabio Andrea, Dobrowolski Radek (2018). TFEB-driven endocytosis coordinates MTORC1 signaling and autophagy. Autophagy.

[39] Neuzillet C, Tijeras-Raballand A, Cohen R, Cros J, Faivre S, Raymond E (2015). Targeting the TGFbeta pathway for cancer therapy. Pharmacol Ther.

[40] Mendoza P, Ortiz R, Diaz J, Quest AF, Leyton L, Stupack D (2013). Rab5 activation promotes focal adhesion disassembly, migration and invasiveness in tumor cells. J Cell Sci.

[41] Frittoli E, Palamidessi A, Marighetti P, Confalonieri S, Bianchi F, Malinverno C (2014). A RAB5/RAB4 recycling circuitry induces a proteolytic invasive program and promotes tumor dissemination. J Cell Biol.

