# Supplementary figures and images for: TFEB-driven autophagy potentiates TGF-β induced migration in pancreatic cancer cells
Source: J Exp Clin Cancer Res. 2019 Aug 6;38:340. doi: 10.1186/s13046-019-1343-4 (PMC6683473; doi:10.1186/s13046-019-1343-4)

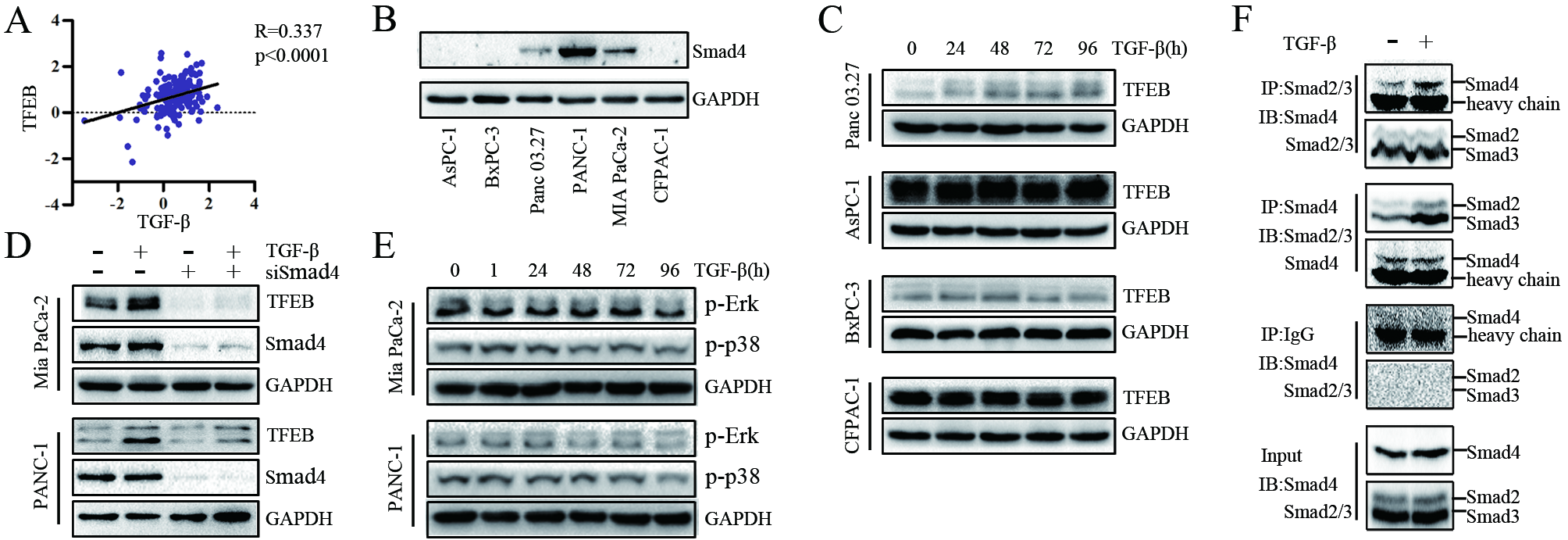

Supplement: Supplementary file 1 — Figure S1A. Analysis of expression correlation based on a TCGA dataset of 184 pancreatic cancer patients. Figure S1B. Western blot showing Smad4 levels in indicated PC cell lines. Figure S1C. Western blot revealing TFEB protein expression in Panc03.27, AsPC-1, BxPC-3 and CFPAC-1 cells by treatment with TGF-β (10 ng/mL) for the times indicated. Figure S1D. Western blot revealing TFEB and Smad4 by treatment with TGF-β (10 ng/mL, 48hous) or/and pretreated with siSmad4 in MIA PaCa-2 and PANC-1 cells. Figure S1E. Western blot showing phosphorylated p44/42 MAP kinase (p-Erk) and phosphorylated p38 MAP kinase (p-p38) expression in MIA PaCa-2 and PANC-1 cells by treatment with TGF-β (10 ng/mL) for the times indicated. Figure S1F. Co-immunoprecipitation analysis of Smad2/3-Smad4 heteromeric complex formation treated with TGF-β (10 ng/mL, 1 h) in PANC-1 cells. (TIF 1532 kb) [file 13046_2019_1343_MOESM1_ESM.tif]

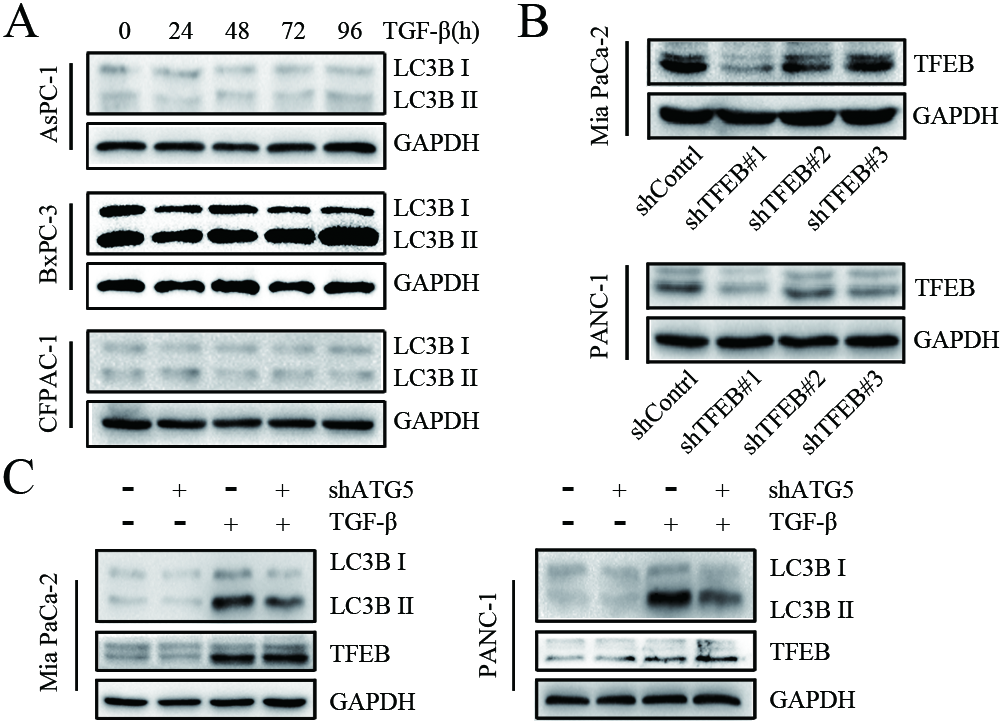

Supplement: Supplementary file 2 — Figure S2A. Western blot showing LC3B expression in AsPC-1, BxPC-3 and CFPAC-1 cells by treatment with TGF-β (10 ng/mL) for the times indicated. Figure S2B. Western blot revealing the efficiency of shRNA targeting TFEB in MIA PaCa-2 and PANC-1 cells. Figure S2C. Western blot showing LC3B and TFEB in MIA PaCa-2 and PANC-1 cells by transfected with shATG5 and/or treated with TGF-β (10 ng/mL, 48 h). (TIF 1039 kb) [file 13046_2019_1343_MOESM2_ESM.tif]

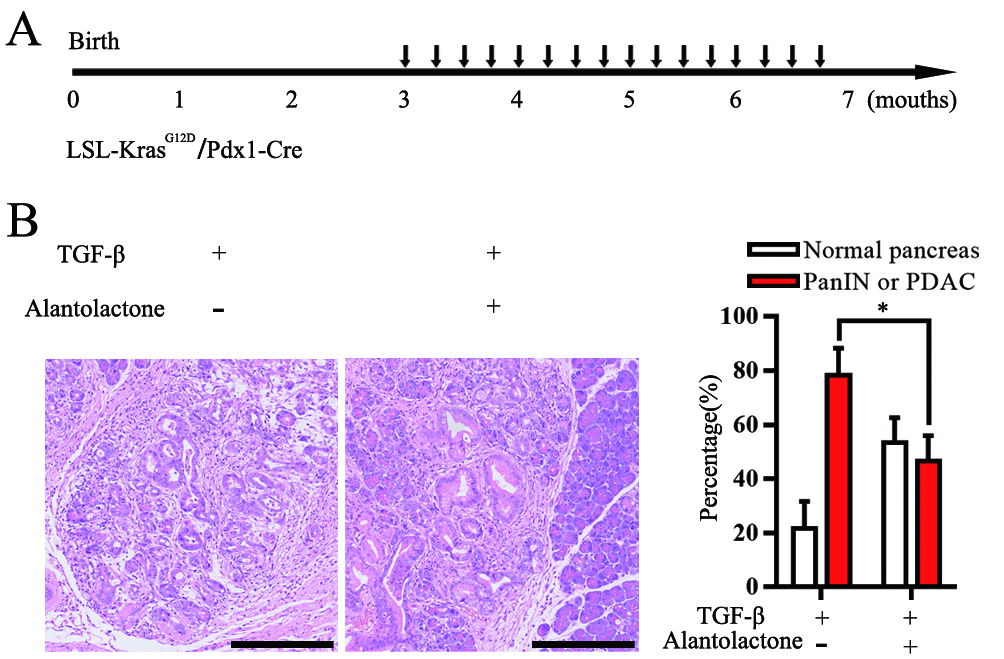

Supplement: Supplementary file 3 — Figure S3A. The schematic illustration shows injection of TGF-β with or without alantolactone in LSL-KrasG12D/Pdx1-Cre mice model. Arrowhead: injection frequency. Figure S3B. Pancreatic pathology images reveal representative 7-month-old LSL-KrasG12D/Pdx1-Cre mice by treatment of TGF-β with or without alantolactone. Quantification of the percentage of normal ducts and PanIN or PDAC is shown in the right panel. (TIF 1274 kb) [file 13046_2019_1343_MOESM3_ESM.tif]

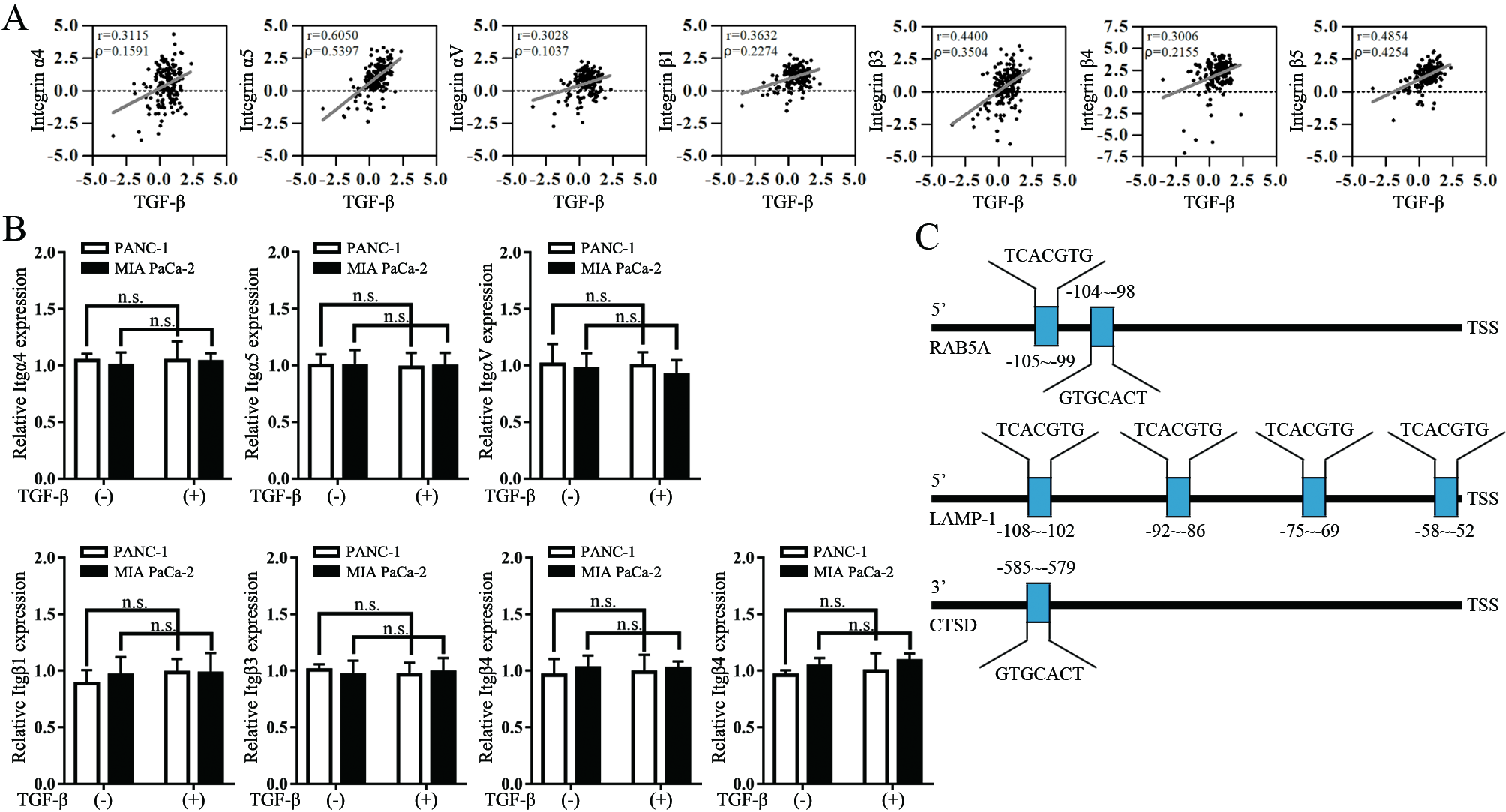

Supplement: Supplementary file 4 — Figure S4A. Analysis of expression correlation as indicated based on a TCGA dataset of 184 pancreatic cancer patients. Figure S4B. Real-time PCR showing relative mRNA level of indicated integrin in PC cells. Figure S4C. TCACGTG motif analysis in promoter sequence of RAB5A, LAMP-1 and CTSD gene in Homo sapiens. (TIF 1313 kb) [file 13046_2019_1343_MOESM4_ESM.tif]

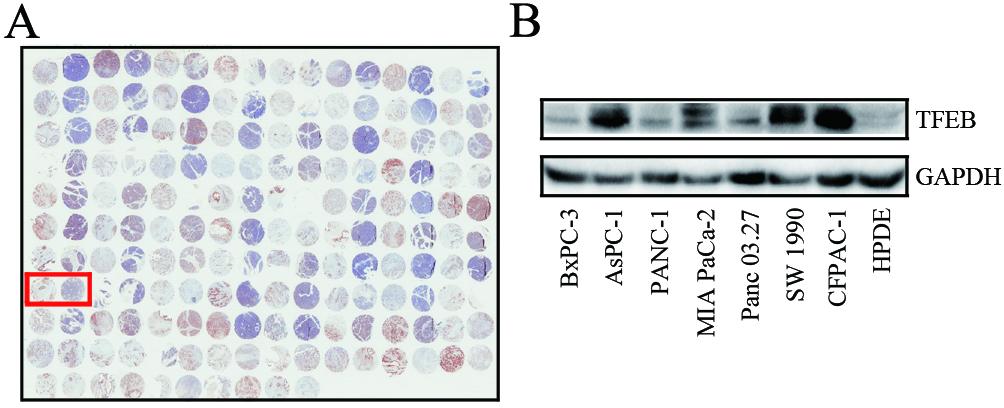

Supplement: Supplementary file 5 — Figure S5A. Immunohistochemical analysis shows image of TFEB expression in PC tissues. Figure S5B. Western blot showing TFEB levels in indicated PC cell lines. (TIF 1090 kb) [file 13046_2019_1343_MOESM5_ESM.tif]
